# Molecular Characterization of *msp2/p44* of *Anaplasma phagocytophilum* Isolated from Infected Patients and *Haemaphysalis longicornis* in Laizhou Bay, Shandong Province, China

**DOI:** 10.1371/journal.pone.0078189

**Published:** 2013-10-22

**Authors:** Yong Wang, Chuangfu Chen, Lijuan Zhang

**Affiliations:** 1 College of Animal Science & Technology, Shihezi University, Shihezi, Xinjiang Province, People’s Republic of China; 2 Department of Rickettsiology, National Institute for Communicable Disease Control and Prevention, China CDC, Beijing, People’s Republic of China; National Center for Biotechnology Information (NCBI), United States of America

## Abstract

Molecular characterization of the MSP2/P44 protein of *Anaplasma phagocytophilum* may determine not only if the bacterium is capable of invading hosts but also whether it generates antigenic variation for the purpose of escaping the host immune response, resulting in various pathologic injuries and serious clinical outcomes. Chinese anaplasmosis patients usually present with serious manifestations, and the fatality rate is as high as 26.5%. In this study, we amplified, cloned and sequenced the *msp2/p44* genes of three Chinese *A. phagocytophilum* isolates from Laizhou Bay, Shandong Province, where human granulocytic anaplasmosis (HGA) patients present severe clinical manifestations, and analyzed their genetic characterization and structural features. We also compared them with the HZ and Webster *A. phagocytophilum* strains. The sequences for both strains are available in GenBank. Analyses indicated that Chinese *A. phagocytophilum* isolates were significantly different from the HZ and Webster strains in terms of nucleotide sequences, amino acid sequences and protein secondary and tertiary structures. Moreover, the number of immunologic B-cell epitopes (19) of the MSP2 protein of the Chinese isolates was higher than that of the *A. phagocytophilum* strains HZ (16) and Webster (9). This genetic diversity of the MSP2/P44 protein of Chinese *A. phagocytophilum* isolates might be relevant and might have serious clinical outcomes. This observation could provide a clue to further understand the pathogenesis of Chinese *A. phagocytophilum*.

## Introduction

*Anaplasma* (*A*) *phagocytophilum* (APH) is a Gram-negative and obligate intracellular pathogen that infects mammal hosts worldwide and is transmitted by ticks [1–3]. *A. phagocytophilum* is an important zoonotic pathogen in that it infects not only humans but also some domestic animals, including horses, dogs, cattle and sheep [4,5]. The life cycle of APH is related to its natural hosts, such as rodents and ruminants, as well as its transmission vectors, which include some members of the genera *Ixodes* and *Haemaphysalis* [6-12]. Humans are a dead-end host for *A. phagocytophilum* and are thus not part of the life cycle of the bacteria [13]. The transmission of *A. phagocytophilum* into mammals mainly relies on infected tick vectors. However, in 2006, the nosocomial transmission of *A. phagocytophilum* was proven in the Anhui Province in China, suggesting that the pathogen was transmitted through contact with blood or respiratory aerosols from infected patients [14]. The manifestations of human granulocytic anaplasmosis (HGA) infection include fever, chills, headache, myalgia, leukopenia and thrombocytopenia, as well as elevated levels of liver aminotransferase [15]. The number of HGA cases has increased annually because of certain natural and social factors, such as global warming, increases in outdoor activities, globalization of the economy and worldwide trade. The number of *A. phagocytophilum* infection cases reached 1,161 in the United States in 2009, and the HGA case-fatality rate in the Midwestern United States is 0.6%- 0.7%. This rate may be on the rise, however, due to misdiagnosis [13]. Additionally, tick-borne ruminant fever (for example, cattle and sheep) caused by *A. phagocytophilum* infection is common in Europe [16]. In Asian countries, including China, Japan and South Korea, HGA cases and HGA agents have been continuously discovered and detected in the last few years [7-12,17-20]. 

A growing number of medical reports indicate that the clinical manifestations of Chinese HGA patients are significantly different than those of patients from Western countries. The HGA occurring in China is usually accompanied by several life-threatening complications, including systemic inflammation response syndrome (SIRS) and multiple organ dysfunction syndrome (MODS). Moreover, the fatality rate of Chinese HGA patients has been reported to be as high as 26.5% [21]. Therefore, studies that genetically characterize virulence factors and examine the pathogenesis of native Chinese *A. phagocytophilum* isolates have major clinical and public health significance in China. The members of the outer membrane protein OMP1/MSP2/P44 superfamily are regarded as important virulence factors of *A. phagocytophilum* pathogens. Genetic variation of the MSP2/P44 protein may not only determine if the bacterium is capable of invading the host but also whether it can generate antigenic variation to allow for escape from the host immune response, resulting in various pathologic injuries and serious clinical outcomes [21-24]. Therefore, *A. phagocytophilum* pathogenesis, which is related to the genetic characteristics of MSP2/P44, has recently become a topic of growing interest. Given the severe clinical manifestations of HGA in China, we focused on the analysis of the genetic variation of the *msp*2*/p44* genes of three native Chinese *A. phagocytophilum* isolates from Laizhou Bay, Shandong Province, where 100% of patients had severe clinical manifestations.

## Materials and Methods

### Ethics statement

The use of pathogenic DNA isolated from patients was approved by the ethics committee of the Chinese CDC (No. 201103), and all samples were anonymized.

### Bacteria strains

Three native Chinese *A. phagocytophilum* isolates, including two human isolates (LZ-HGA-agent-3 and LZ-HGA-agent-4) from HGA patients and one tick isolate (named LZ-HGA-agent-T1) from infected *Haemaphysalis* (*H*) *longicornis*, were isolated at Laizhou Bay in Shandong Province in 2009 - 2010. All three pathogenic isolates were cultured and conserved in HL-60 cell lines in our laboratory. The two human pathogens were isolated from patients with severe clinical manifestations; the *ank A* genes from the samples had 100% identity with each other and were 100% homologous to the tick isolate (LZ-HGA-agent-T1) [25]. 

### PCR primer design

**Figure 1:**
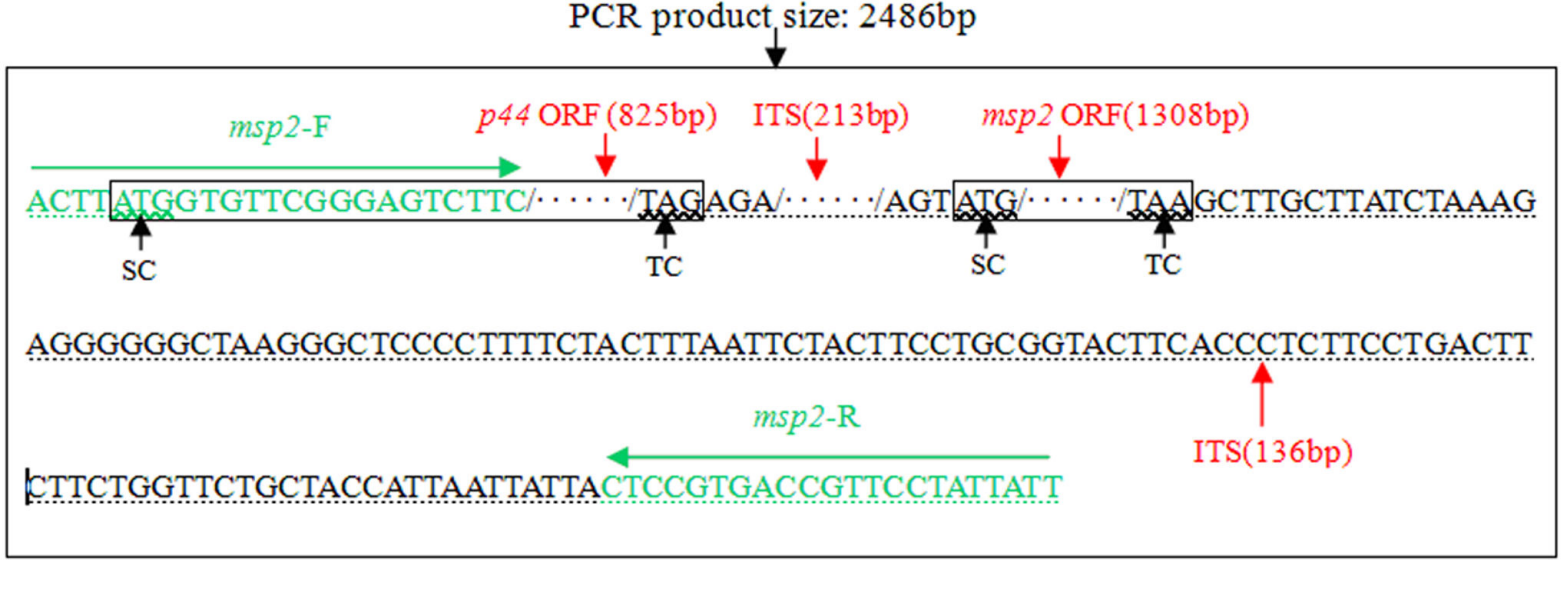
Sequences and positions of the *msp2/p44* PCR primers and the predicted size of the PCR product. The PCR product and its size are shown in the larger box. The two smaller boxes inside the larger box indicate the *p44* ORF and *msp2* ORF, respectively. Inter-genic sequences (ITS) are shown using dotted lines under letters. Ellipses inside the PCR product indicate the omission of some letters. The PCR products of the primers *msp2*-F and *msp2*-R are indicated using green letters under the green arrowhead. SC: start codon; TC: terminal codon.

The *msp*2*/p44* genes of *A. phagocytophilum* usually contain two open reading frames (ORFs) with *msp2* and *p44* [26,27]. To obtain *msp*2*/p44*, PCR primers were initially designed with the bio-software Primer Premier 5.0, according to the Webster strain sequence (accession number AY164491) of *A. phagocytophilum*, published in the GenBank database. The specificity of the PCR primers was also assessed using an online program (http://www.ncbi.nlm.nih.gov/Tools/primer-blast/). The names and relative sites of the PCR primers and the predicted size of the PCR products are shown in Figure 1. 

### PCR amplification analyses and sequencing

Genomic DNA (gDNA) was separately prepared from three Chinese native *A. phagocytophilum* isolates (LZ-HGA-agent-3, LZ-HGA-agent-4 and LZ-HGA-agent-T1) using a DNeasy^®^ Blood & Tissue Kit (QIAGEN, Cat No. 69506) and was then used as a PCR template. The PCR primers were as follows: *msp2*-F 5’-ACTTATGGTGTTCGGGAGTCTTC-3’ and *msp2*-R 5’-AATAATAGGAACGGTCACGGAG-3’, and the predicted size of the PCR product was 2,486 bp. Briefly, 3.0 μL of gDNA was used as a template in a 25-μL reaction mixture system containing 2.5 μL 10×*Taq* Buffer (SDS Genetech Co., Ltd, China, Cat# ET-500), 1.0 μL of each primer: *msp*2-F and *msp*2-R (0.4 μM final concentrations of each primer), 0.5 μL of deoxynucleoside triphosphates (dNTPs, 10 mM), 2.5 μL of dye, 0.5 μL of Taq DNA polymerase (5 U/μL, SBS Genetech Co., Ltd, China, Lot#042512) and 14 μL of ddH_2_O. PCR was performed using a SensoQuest LabCycler standard plus (SensoQuest GmbH, Goettingen, Germany) with a pre-denaturation at 94°C for 5 min, followed by 35 cycles of a denaturation step at 94°C for 40 seconds, an annealing step at 57°C for 40 seconds and an extension step at 72°C for 3 minutes. There was a final extension at 72°C for 10 minutes. The PCR amplification products were analyzed using 1.0% agarose gel electrophoresis. To obtain the entire sequences of the *msp*2*/p44* genes and to avoid the loss of some sequence information at the ends of both primers, we cloned the PCR products as follows: the PCR product was purified using a multi-function DNA purification kit (BioTeke Corporation, Cat#DP1502). Purified *msp2/p44* was cloned into a *pEASY*-T1 Cloning vector (Beijing TransGen Biotech Co., Ltd., Lot#G30716), and the recombinant plasmid was designated *pEASY*-*msp2/p44*. The recombinant plasmid *pEASY*-*msp2/p44* was transformed into *E. coli* DH5a competent cells. Positive clones were screened by PCR using the primers *msp2*-F and *msp2*-R. The recombinant plasmid was extracted from positive clones using a high-purification plasmid mini-preparation kit (BioTeke Corporation,Cat#DP1002) and then directly sequenced by two separate commercial sequencing companies in China: Beijing Tsingke BioTech Co., Ltd. and Sangon BioTech (Shanghai) Co., Ltd. 

### Data analysis

The sequencing was performed with universal primers from the *pEASY*-T1 Cloning Kit (Beijing TransGen Biotech Co., Ltd., Lot#G30716) and using the Sanger sequencing method. The sequences of *msp2/p44* were processed through manual splicing and proofreading and were also analyzed using the nucleotide blast program (http://blast.ncbi.nlm.nih.gov/). For the analysis of the *msp2/p44* sequences, the DNASTAR package (Lasergene, Madison, WI) was used. The *msp2/p44* nucleotide sequences and their coded amino acid sequences were edited with the EditSeq program of the package. The *msp2/p44* nucleotide sequences and their coded amino acid sequences were then aligned with the MegAlign program of the package by comparison with the corresponding sequences from the *A. phagocytophilum* HZ and Webster strains. For the purpose of delineating genetic evolution information of the native Chinese *A. phagocytophilum* isolates, a phylogenetic tree was constructed with 10 sequences, including Chinese *A. phagocytophilum* isolates and another 9 varying *Anaplasma* strains (Table 1), which were identified in different hosts from different geographic regions. The msp2 sequences for *A. phagocytophilum* HZ (CP000235) and Webster (AY164491) were used for outgroup comparisons. The phylogenetic analysis of the *msp2/p44* gene sequences was conducted using the program MEGA 5.05 (Arizona State University), as previously described [6]. In general, the sequences were aligned using CLUSTAL W of MEGA 5.05, with the application of the IUB matrix for nucleotide sequences and the Gonnet matrix for protein sequences. Tree construction was achieved using the neighbor-joining method with the complete deletion option, using the Jukes-Cantor matrix for nucleotide sequences and the Dayhoff matrix for protein sequences. Bootstrap analysis was conducted with 1,000 replicates. 

**Table 1:** Selected *msp2/p44*(*p44ESup1/omp-1*) gene sequences for the phylogenetic analysis.

**Accession No.**	**Bacteria strains**	**Hosts**	**Geographic origin**	**Remark**
KC128828/KC430333/KC430334	LZ-HGA-Agent	human	China	*p44*+*msp2*
CP000235	APH-HZ	human	USA	*Omp-1N*+*msp2*
AY164491	APH-Webster-var A	human	USA	*p44ESup*1+*msp2*
AY164492	APH- HGE2-var II1	human	USA	*p44ESup*1+*msp2*
AY164493	APH	human	USA	*p44ESup*1+*msp2*
AY137510	APH-NY-37	human	USA	p44+*omp*-1
FJ600595	APH-Tick-176-5ES-Iwate-Ip	*Ixodes persulcatus*	Japan	p44+*omp*-1
FJ600601	APH-Tick-176-5ES-Iwate-Ip	*Ixodes persulcatus*	Japan	p44+*omp*-1
DQ519565	APH-NORSHES	sheep	Norway	*p44ESup*1+*msp2*
DQ519566	APH-SWDOGES	dog	Sweden	*p44ESup*1+*msp2*

Note: *omp-*1 may stand for *omp-*1N, *omp-*1X or both; APH: *A. phagocytophilum*

### Bioinformatics analysis of the MSP2/P44 protein

The structural information for the MSP2/P44 protein was predicted and delineated using online software and/or programs. In particular the ProtParam tool (http://web.expasy.org/protparam/) was used for the primary structure of the protein and the Predict Secondary Structure (PSIPRED v3.0) (http://bioinf.cs.ucl.ac.uk/psipred/) for the secondary structures. TMHMM Server v.2.0 (http://www.cbs.dtu.dk/services/TMHMM/) was used for the transmembrane domains, BepiPred 1.0 Server (http://www.cbs.dtu.dk/services/BepiPred/) was used for B-cell epitope-bearing regions, and Galaxy TBM (http://galaxy.seoklab.org/) was used for the protein tertiary structure prediction from sequences obtained with template-based modeling. 

Accession numbers of the full-length *msp2/p44* gene sequences of LZ-HGA-Agent-3, LZ-HGA-Agent-4 and LZ-HGA-Agent-T1 have been deposited in the GenBank database under the accession numbers KC128828, KC430333 and KC430334, respectively.

## Results

### PCR amplification and msp2/p44 gene sequencing

PCR amplification revealed that the predicted 2.5-kb fragments of the *msp2/p44* genes were successfully amplified, using the *msp*2-F/*msp*2-R primer pair and the genomic DNA of three native Chinese *A. phagocytophilum* isolates, namely LZ-HGA-Agent-3 (KC128828), LZ-HGA-Agent-4 (KC430333) and LZ-HGA-Agent-T1 (KC430334), as templates. The sequencing analysis indicated that the sequences of *msp2/p44* in all of the isolates were 100% identical to each other at the nucleotide level and were mainly composed of one *p44* (825 bp) open reading frame (ORF), one *msp2* (1323 bp) ORF and a few intergenic sequences (ITS) (Figure 1). Therefore, the three native Chinese *A. phagocytophilum* isolates mentioned above were designated LZ-HGA-Agent (KC128828/KC430333/KC430334) for simplifying the description in the study, where the name collectively stands for LZ-HGA-Agent-3, LZ-HGA-Agent-4 and LZ-HGA-Agent-T1. 

The *p44* ORF sequence of LZ-HGA-Agent is 100% and 99.6% identical to the *p44ESup1* sequence of the Webster strain of *A. phagocytophilum* and the *omp-1N* sequence of the HZ strain, respectively. There is a 3-bp difference in the *p44* ORF sequence of LZ-HGA-Agent compared with the *omp-1N* sequence of the HZ strain at the nucleotide level. The details of the differences are as follows: A to G nucleotide sequence change at positions 270 and 720 and G to A at position 484 in the HZ strain sequence (data not shown). In contrast, the *msp2* ORF sequence of LZ-HGA-Agent displays 87.7% and 45.1% identity to that of the *msp2* sequences of both the *A. phagocytophilum* Webster and HZ strains, respectively. The LZ-HGA-Agent *msp2* ORF sequence displays relatively little homology to the *msp2* sequences of the *A. phagocytophilum* HZ strain (45.1%) when contrasted with the *A. phagocytophilum* Webster strain (87.7%) because of the occurrence of large-scale diversity at the nucleotide level (see Figure S1). 

The P44 amino acid sequence, which is encoded by the *p44* ORF in the LZ-HGA-Agent, is 100% and 99.6% similar to the *p44ESup1*-coded product sequence of the Webster strain and the *omp-1N*-coded product sequence of the HZ strain at the amino acid level, respectively. The LZ-HGA-Agent and the HZ strains have identical P44 sequences, except for a difference in the amino acid at position 162, namely V to M in the HZ strain sequence (data not shown). The MSP2 amino acid sequence, which is encoded by *msp2* ORF in LZ-HGA-Agent, displays 84.6% and 27.9% homology to the MSP2 amino acid sequences of the *A. phagocytophilum* Webster and HZ strain, respectively. It is of note that, for the *msp2* ORFs and the coding amino acid sequences that were analyzed in this work, the identities and similarities between the different *msp2* sequences in various strains demonstrate that coding amino acid similarities are lower than nucleotide identities, suggesting that the *msp2* nucleotide exchanges of LZ-HGA-Agent were extremely nonsynonymous substitutions (see Figure S1 and Figure S2). Thus, we conclude that extreme differences in the genetic variation of the *msp2* ORF sequence and its amino acid sequence in LZ-HGA-Agent exist, but such extreme differences do not exist with the *p44* ORF sequences. 

In this study, we also compared our results with another Chinese sequence of *msp2/p44* (EU 008082) identified in rodents in the southeast of China [28], and the results indicated that the identity of the *msp2* nucleotide sequence (from nt 1475 to 2352) and the amino acid sequences of both the *A. phagocytophilum* LZ-HGA-Agent strain and the rodent (EU008082) strain were 48.4% and 27.7%, respectively.

### Phylogenetic analysis

**Figure 2:**
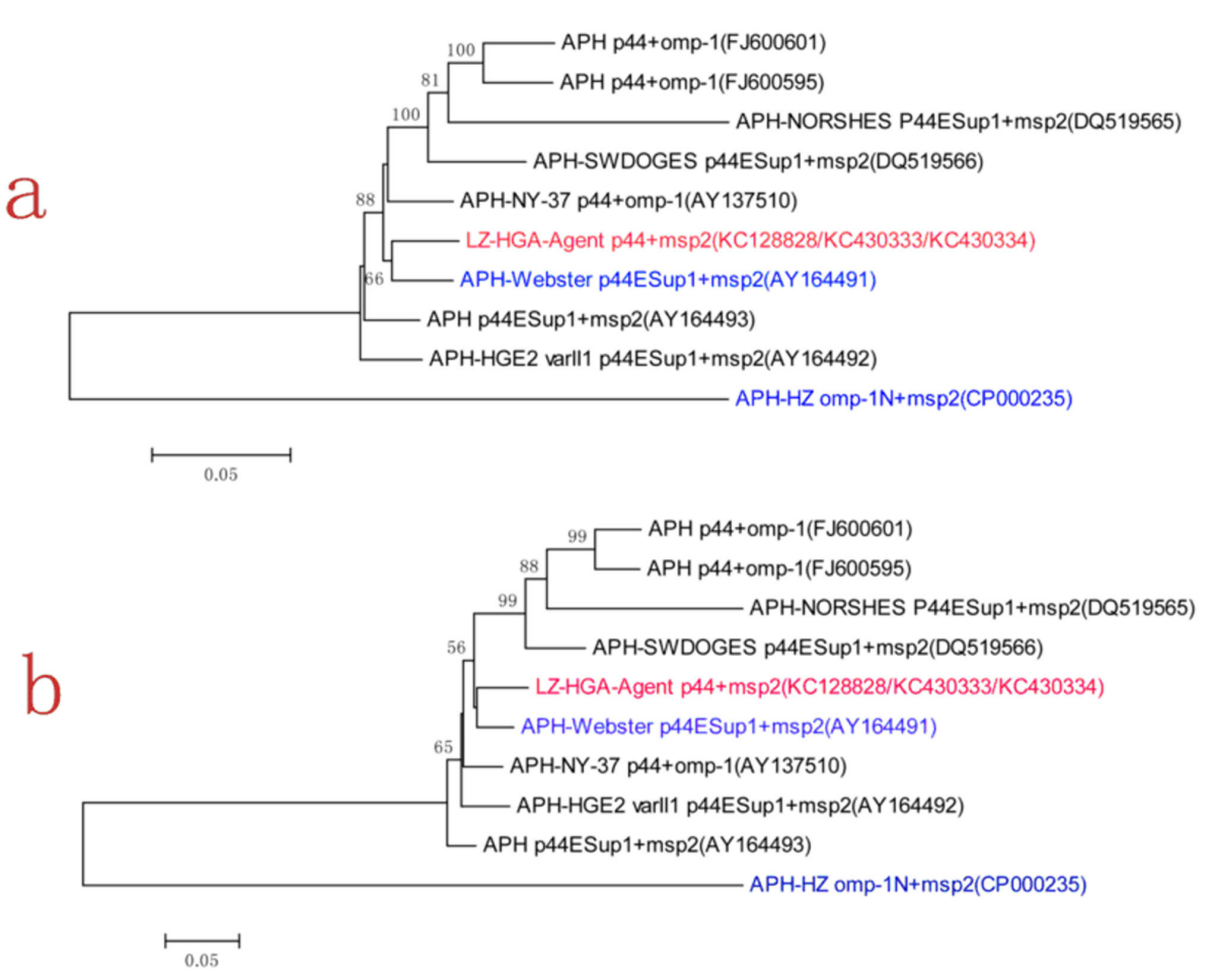
Phylogenetic tree based on the *msp2/p44* nucleotide sequences (a) and MSP2/P44 amino acid sequences (b) generated using the neighbor-joining method. **a**. Bootstrap values (>60%) are shown next to the nodes of the tree, and the scale bar indicates the number of nucleotide substitutions per site; **b**. The MSP2/P44 amino acid sequences were obtained by *msp2/p44* gene sequence translation, and the bootstrap values (>50%) are shown next to the nodes of the tree. The scale bar indicates the number of amino acid substitutions per site. The Chinese isolate LZ-HGA-Agent (red) and the international reference strains for APH-Webster and APH-HZ (blue) are highlighted. APH: *Anaplasma phagocytophilum*.

To assess the relationship between LZ-HGA-Agent and other strains of *A. phagocytophilum* investigated in this study, another 9 sequences identified in different host species from different geographic regions were used to construct a phylogenetic tree (Table 1). Specifically, we constructed a neighbor-joining (NJ) tree. As shown in Figure 2a, all major branches referring to the gene sequences used in the work were supported by bootstrap values >60%. Using amino acid sequences to construct the tree, similar results were obtained (Figure 2b). From Figure 2a and b, we noticed that the *A. phagocytophilum* Chinese isolate LZ-HGA-Agent (KC128828/KC430333/KC430334) was very closely related to the human *A. phagocytophilum* Webster strain (AY164491) from the United States, human strain NY-37 (AY137510) from the United States, sheep NORSHES strain (DQ519565) from Norway, canine NORSHES strain (DQ519566) from Sweden and tick strain Tick-176-5ES-Iwate-Ip (FJ600595 and FJ600601) from Japan, but was less related to the human HZ strain (CP000235) from the United States (Figure 2a and 2b). 

### Bioinformatics analyses of the MSP2 protein

Because the *p44* ORF sequence of LZ-HGA-Agent displayed a higher homology to the *p44ESup1* sequence (100%) of *A. phagocytophilum* Webster (APH-Webster) and the *omp-1N* sequence (99.6%) of *A. phagocytophilum* HZ (APH-HZ), their bioinformatics analyses were not conducted in detail in this study. In contrast, we focused on the analyses of the MSP2 proteins of the different strains.

### MSP2 amino acid composition

The amino acid composition of the MSP2 proteins belonging to LZ-HGA-Agent, APH-HZ and APH-Webster were analyzed using the ProtParam tool (http://web.expasy.org/protparam/), and the results are shown in Table 2. The more abundant amino acids in MSP2 from LZ-HGA-Agent include Gly (12.5%), Ala (9.5%) and Val (9.5%), and the percent of the Gly content (12.5%) of LZ-HGA-Agent-MSP2 was higher than that of APH-HZ-MSP2 (10.1%) but lower than that of APH-Webster-MSP2 (13.8%). The Val content (9.5%) of LZ-HGA-Agent-MSP2 was nearly equal to that of APH-HZ-MSP2 (8.8%) and APH-Webster-MSP2 (9.7%). The percentage of the Ala content (9.5%) of LZ-HGA-Agent-MSP2 was roughly equal to that of APH-Webster-MSP2 (9.2%) but was obviously higher than that of APH-HZ-MSP2 (5.8%). The MSP2 isoelectric point analysis (pI) indicated that the pI (5.87) of LZ-HGA-Agent-MSP2 from China was approximately equal to the pI (5.59) of human APH-Webster-MSP2 from the United States but was different than the pI (9.20) of human APH-HZ-MSP2 from the United States. 

**Table 2:** Amino acid composition of MSP2 proteins from LZ-HGA-Agent, APH-HZ and APH-Webster.

	**LZ-HGA-Agent**		**APH-HZ**		**APH-Webster**	
**Amino acid**	**Number**	**Percentage**	**Number**	**Percentage**	**Number**	**Percentage**
Ala (A)	42	9.50%	21	5.80%	40	9.20%
Arg (R)	16	3.60%	22	6.00%	16	3.70%
Asn (N)	18	4.10%	17	4.70%	17	3.90%
Asp (D)	31	7.00%	13	3.60%	32	7.40%
Cys (C)	4	0.90%	2	0.50%	4	0.90%
Gln (Q)	6	1.40%	9	2.50%	7	1.60%
Glu (E)	19	4.30%	22	6.00%	23	5.30%
Gly (G)	55	12.50%	37	10.10%	60	13.80%
His (H)	7	1.60%	9	2.50%	5	1.10%
Ile (I)	17	3.90%	21	5.80%	17	3.90%
Leu (L)	31	7.00%	42	11.50%	30	6.90%
Lys (K)	29	6.60%	20	5.50%	33	7.60%
Met (M)	10	2.30%	9	2.50%	10	2.30%
Phe (F)	16	3.60%	19	5.20%	15	3.40%
Pro (P)	16	3.60%	9	2.50%	12	2.80%
Ser (S)	32	7.30%	27	7.40%	27	6.20%
Thr (T)	29	6.60%	17	4.70%	25	5.70%
Trp (W)	2	0.50%	2	0.50%	2	0.50%
Tyr (Y)	18	4.10%	15	4.10%	18	4.10%
Val (V)	42	9.50%	32	8.80%	42	9.70%

#### MSP2 secondary structure

The secondary structures of each MSP2 protein were predicted using the program Predict Secondary Structure (PSIPRED v3.0) (http://bioinf.cs.ucl.ac.uk/psipred/), and the results are shown in Figure S3, Figure S4 and Figure S5. As shown in these figures, all MSP2 protein structures from LZ-HGA-Agent, *A. phagocytophilum* Webster and *A. phagocytophilum* HZ included a main random coil structure, a β-strand structure dispersed to the two ends of the protein and a few α-helices. However, there was a greater number of α-helices in the LZ-HGA-Agent-MSP2 protein than in APH-HZ-MSP2 and APH-Webster-MSP2. In particular, there were six α-helices in the LZ-HGA-Agent-MSP2 from China but only three α-helices in the APH-HZ-MSP2 and five α-helices in APH-Webster-MSP2 from the United States. In contrast, there were 17 β-strands in the LZ-HGA-Agent-MSP2 protein but 20 in APH-HZ-MSP2 and 17 in APH-Webster-MSP2, suggesting that the secondary structure of the LZ-HGA-Agent-MSP2 protein from the Chinese isolate was obviously distinct from the structures of APH-HZ-MSP2 from the United States but not from APH-Webster-MSP2 from the Unites States. 

### MSP2 transmembrane domains

**Figure 3:**
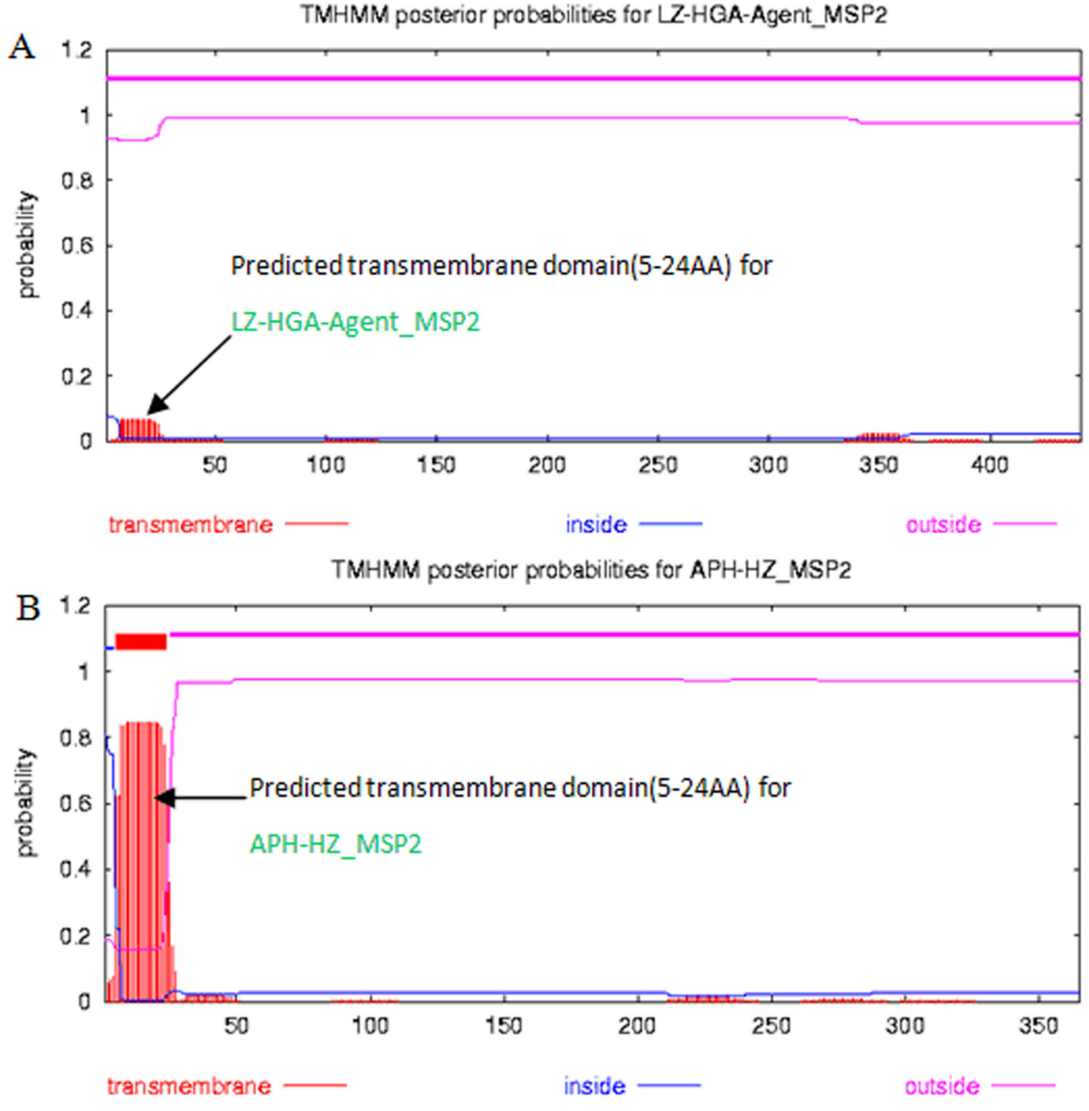
Predicted transmembrane domain for LZ-HGA-Agent-MSP2 (A), APH-HZ-MSP2 (B) and APH-Webster-MSP2 (C). The red legend (transmembrane —), blue legend (inside —) and pink legend (outside —) in panels A, B and C indicate the transmembrane domain, interior domain and exterior domain, respectively, for the MSP2 protein as predicted by the TMHMM program. The number on the horizontal abscissa in panels A, B and C indicates the amino acid (AA) residual site and size.

The transmembrane domains of each MSP2 protein were predicted using TMHMM Server v.2.0 (http://www.cbs.dtu.dk/services/TMHMM/) (Figure 3). The predicted results indicated that the proteins were hardly different in terms of the location of the transmembrane domains of MSP2, when comparing the LZ-HGA-Agent isolate, APH-HZ and APH-Webster strains. The transmembrane domains of MSP2 were basically located in the 5^th^ to 24^th^ amino acid sequence region. 

### B-cell epitope analysis of MSP2

B-cell epitope-bearing regions of each protein were predicted using BepiPred 1.0 Server (http://www.cbs.dtu.dk/services/BepiPred/), and the results are shown in Table 3. Nineteen B-cell epitopes were predicted (the number of amino acids ≥3 at an epitope) in the MSP2 protein of the LZ-HGA-Agent isolate, which was higher than that of the MSP2 proteins from both the APH-HZ and APH-Webster strains, which only had 16 and 9 predicted B-cell epitopes (the number of amino acids ≥3), respectively. 

**Table 3:** Predicted epitopes for LZ-HGA-Agent-MSP2, APH-Webster-MSP2, and APH-HZ-MSP2.

**No.**	**Start/End position**	**Oligo peptide for epitope**	**Peptide length**
1	23/38( LZ-HGA-Agent)	DVRAHDDVSALDTGGA	16
	23/38(APH-HZ)	DVRAHDDVSALETGGA	16
	21/30( APH-Webster)	TSAHADNDKS	10
2	48/50( LZ-HGA-Agent)	SPA	3
	48/50(APH-HZ)	SPA	3
	50/56( APH-Webster)	IDDGGET	7
3	60/68( LZ-HGA-Agent)	RESNGETKA	9
	60/68(APH-HZ)	RESNGETKA	9
	77/83( APH-Webster)	YWGPEVA	7
4	73/76( LZ-HGA-Agent)	LKDG	4
	73/76(APH-HZ)	LKDG	4
	92/98( APH-Webster)	NTTFGGS	7
5	78/80( LZ-HGA-Agent)	SVK	3
	78/80(APH-HZ)	SVK	3
	125/132( APH-Webster)	HKGRKGGG	8
6	86/96( LZ-HGA-Agent)	FDWNTPDPRIG	11
	85/96(APH-HZ)	KFDWNTPDPRIG	12
	191/193 ( APH-Webster)	LKR	3
7	109/115( LZ-HGA-Agent)	VGYGIGG	7
	108/114(APH-HZ)	SVGYGIG	7
	205/209( APH-Webster)	PRNRS	5
8	132/144( LZ-HGA-Agent)	IRDSGSKEDGADT	13
	131/143(APH-HZ)	GIRDSGSKEDEAD	13
	273/276( APH-Webster)	CAGI	4
9	158/164( LZ-HGA-Agent)	TGQTDNL	7
	157/163(APH-HZ)	VTGQTDK	7
	335/345( APH-Webster)	DISPTNSVREK	11
10	170/175( LZ-HGA-Agent)	KTSGKD	6
	169/174(APH-HZ)	AKTSGK	6
11	185/193( LZ-HGA-Agent)	VSHPTIDGK	9
	184/239(APH-HZ)	GVSHPGIDKKVCDGGHARGKKSGDNGSLADYTDGGASQTNKTAQCSGMGTGKAGKR	56
12	196/210( LZ-HGA-Agent)	RTKNGHSTPTTLTAY	15
	250/282(APH-HZ)	TKVGEGKNWPTGYVNDGDNVNVLGDTNGNAEAV	33
13	214/226( LZ-HGA-Agent)	AVESDVKTGNNNN	13
	289/296(APH-HZ)	ELTPEEKT	8
14	233/246( LZ-HGA-Agent)	AGSTDGTGSSSPQV	14
	306/309(APH-HZ)	IEGG	4
15	257/287( LZ-HGA-Agent)	GDGSKNWPTSTLKAGGSNGPTPVHNDNAKAV	31
	392/398(APH-HZ)	GVYDDLP	7
16	295/302( LZ-HGA-Agent)	LTPEEKTI	8
	402/415(APH-HZ)	LVDDTSPAGRTKDT	14
17	312/315( LZ-HGA-Agent)	EGGE	4
18	398/404( LZ-HGA-Agent)	VYDDLPA	7
19	408/421( LZ-HGA-Agent)	VDDTSPAGRTKDTA	14

APH-HZ: *A. phagocytophilum* HZ strain, APH-Webster: *A. phagocytophilum* Webster strain, LZ-HGA-Agent: Chinese *A. phagocytophilum* isolate.

#### MSP2 tertiary structures

**Figure 4:**
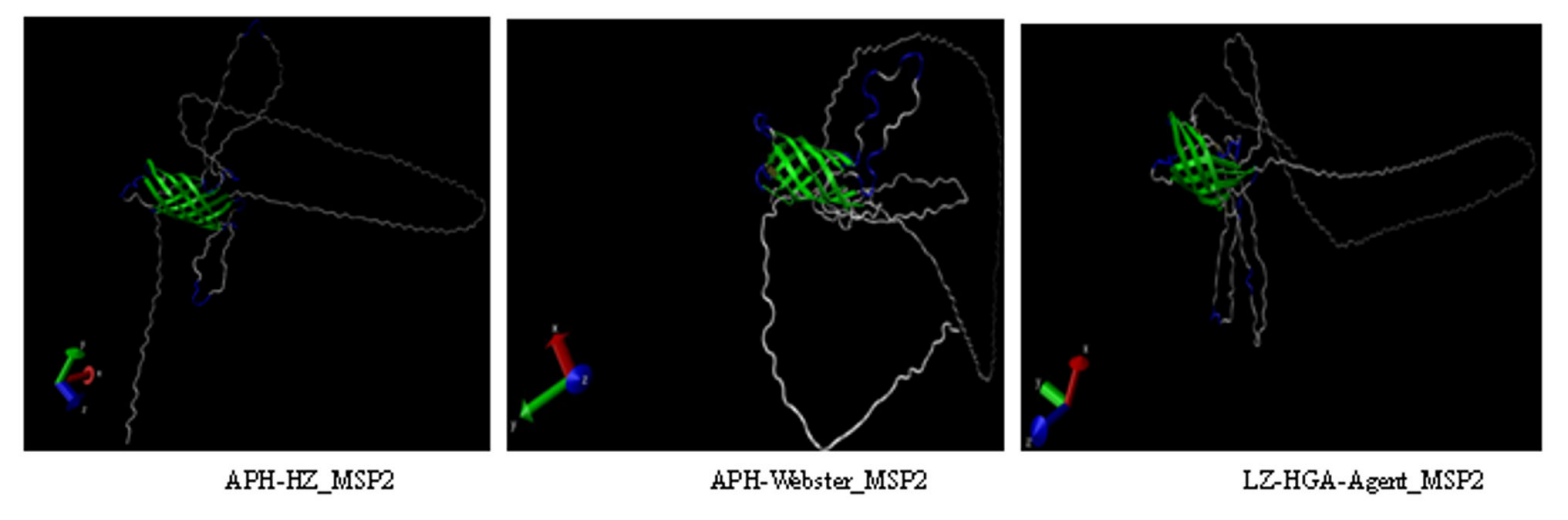
Predicted tertiary structures of MSP2 proteins based on the Nspa (PDB: 1P4T-A) template. The proteins sequences were copied into GalaxyTBM (http://galaxy.seoklab.org/) to compute their tertiary structures. The β-sheet (green), β-turn (blue) and random coil (gray) are highlighted.

Protein tertiary structure prediction was performed using the program GalaxyTBM (http://galaxy.seoklab.org/), and the predicted results are shown in Figure 4. The predicted results indicated that the MSP2 protein tertiary structures of the LZ-HGA-Agent isolate, the APH-HZ and the APH-Webster strains are very different (Figure 4). 

## Discussion

The *A. phagocytophilum* HZ strain was first isolated from a patient with human immunodeficiency disease in New York, USA, in 1995 [29]. This strain caused typical clinical manifestations of HGA and could be cultured by HL60 cells and formed morulas in the cytoplasm of the culture cells. Moreover, this strain is highly cross-reactive with other HGA agents and *E. chaffeensis*. The genomic size of the *A. phagocytophilum* HZ strain is approximately 1.47 Mbps, consisting of 1,369 ORFs, over 100 *p44* (*msp2*) genes, type IV secretion (T4S) and numerous repeats [13,30]. The human HZ strain infects granulocytes by subverting its powerful innate antimicrobial defenses, which also makes infected humans and animals more susceptible to opportunistic infection and causes the resulting endothelial cell adhesion, transmigration, motility, degranulation, respiratory burst and phagocytosis [13]. The changes in these functions are to increase bacterial dissemination into the neutrophil.

The *A. phagocytophilum* Webster (Wisconsin) strain was isolated from a patient in northwestern Wisconsin in 1996 [31], where the seroprevalence of HGA among permanent residents is as high as 14.9% [32], the prevalence of *A. phagocytophilum* in deer was 8.9%-11.5%, and the infection rate in ticks was 5.6%-26% [33]. It is noteworthy that serological cross-reaction assays indicated that there was a striking antigen difference between the *A. phagocytophilum* strains Webster and HZ [34]. 

The LZ-HGA-Agent isolates were isolated from patients and tick-vectors from Laizhou Bay, Shandong Province, during 2009-2010 [25], which is the largest wetland in northern China and a famous migratory bird post across Asia and the West Pacific. As seen in a recent clinical report [21], these two cases were characterized by severe clinical manifestations, including systemic inflammatory response syndrome (SIRS) and multiple organ dysfunction syndrome (MODS). In addition, nearly 100% of Chinese HGA patients in these areas had severe clinical features including SIRS and MODS, significantly lower WBC counts and PLT counts, as well as significantly elevated levels of LDH, CK, BUN, ALT and AST.

The pathogenesis of *A. phagocytophilum* is an issue of worldwide significance because of the effects of the bacteria on human public health. Currently, the members of the major outer membrane protein superfamily OMP-1/MSP2/P44, belonging to *A. phagocytophilum*, are known to be important genetic determinants of pathogenesis and can allow *A. phagocytophilum* to not only adhere to the host cells but also to avoid the host immune surveillance, thus contributing to colonization of the host intracellular environment [4,13,26,35]. The analysis based on comparative genomics demonstrated that the expansion of the *msp2*/*p44* family is a common feature in *A. phagocytophilum* strains [30]. The diversity of paralogous *p44* genes is related to their geographic origin, host-specificity and the mechanisms of functional divergence [36–38]. As a typical example, *p44*-*1* was found in all human isolates from New York State but not in isolates from Minnesota, whereas *p44*-*18* was found in isolates from both regions [36]. The antigenic variability of *msp2*/*p44* is due to differential expression of major immunodominant outer membrane proteins encoded by members of a multigene family [39]. 

The homologous recombination of *p44/msp2* occurs by the use of one of the most important gene conversion mechanisms, the RecFOR recombination pathway, because *A. phagocytophilum* lacks the RecBCD pathway [13,29,35,40,41]. Consequently, the antigenic variation of the P44/MSP2 proteins of *A. phagocytophilum* is most likely an intrinsic property, contributing to the bacterial survival by subverting the host immune system and the persistence within the host intracellular environment [13,29,35,42,43].

In this work, the *msp2* sequences of the Chinese native LZ-HGA-Agent showed a striking difference, both at the nucleotide and amino acid levels (Figure S1 and Figure S2). At the same time, bioinformatics analyses indicated that the Chinese isolates possessed unique protein secondary structures, as the number of α-helices in this strain was greater than that of the proteins in the *A. phagocytophilum* HZ and Webster strains from the United States. However, the MSP2 protein of the LZ-HGA-Agent isolate had fewer β-strands than the HZ strain while maintaining the same number as the Webster strain (see Figures S3, S4 and S5). In addition, a major difference in tertiary structures of the MSP2 protein was observed between the Chinese LZ-HGA-Agent isolate and both the APH-HZ and APH-Webster strains from the USA. The B-cell epitopes in the MSP2 protein of the LZ-HGA-Agent isolate were clearly more prevalent than those in the HZ and Webster strains.

The second and tertiary structures of the MSP2 protein may directly influence its spatial conformations/structures and may change its biological function in terms of host adaptation, bacterial adhesion and bacterial membrane structural integrity. 

B-cell epitopes (also called antigenic determinants) are specific regions that are recognized by and/or interact with immunoreceptors and/or antibody molecules of the B-lymphocyte surface during pathogen-host cell interaction. Multi-antigenic epitopes may exist on one single protein, and various antigenic epitopes may play a different role in bacterial infection. Furthermore, the amount, relative position, stability and conformation of the antigenic epitope are closely related to the protein behavior. The differences in the B-cell epitopes of MSP2 of the LZ-HGA-Agent isolate may directly influence the recognition of immunological B cells by the bacteria, resulting in host cell invasion and, subsequently, the severe clinical symptoms observed following infection with the pathogen isolate in China. 

Although we recognize that three isolates of *A. phagocytophilum* is limited, the LZ-HGA-Agent isolate data obtained in this work may further help us to enhance our basic genetic knowledge of the pathogenesis and biology of the Chinese *A. phagocytophilum* pathogenic strains. The next study will continue to gather more isolates of Chinese *A. phagocytophilum* pathogens and will focus on determining how antigenic variation of the MSP2/P44 protein family contributes to the biology and/or pathogenesis of the human isolate LZ-HGA-Agent and which antigenic variations of the same protein family are involved in the severe clinical symptoms of HGA patients in China.

## Supporting Information

Figure S1 ***msp2* linear alignment of LZ-HGA-Agent, *A. phagocytophilum* HZ and *A. phagocytophilum* Webster at the nucleotide level.** The alignment report was performed using the MegAlign program of the DNASTAR package. The nucleotide sequence names are indicated to the left, and the nucleotide numbers are shown to the right. The solid, deep red letters differ from the consensus, whereas all others match the consensus. APH-Webster: *A. phagocytophilum* Webster strain, APH-HZ: *A. phagocytophilum* HZ strain.
(PDF)

Figure S2 **MSP2 linear alignment of LZ-HGA-Agent, *A. phagocytophilum* HZ and *A. phagocytophilum* Webster at the amino acid level.** The alignment report was performed using the MegAlign program of the DNASTAR package. Amino acid sequence names are indicated to the left, and the amino acid numbers are shown to the right. The amino acid residues colored a solid, deep red differ from the consensus sequence, and all others match the consensus. AA: amino acid residue. APH-Webster: *A. phagocytophilum* Webster strain, APH-HZ: *A. phagocytophilum* HZ strain.
(PDF)

Figure S3 **Putative secondary structure of LZ-HGA-Agent MSP2 determined using the Predict Secondary Structure (PSIPRED v3.0) program** (http://bioinf.cs.ucl.ac.uk/psipred/).
(PDF)

Figure S4 **Putative secondary structure of *A. phagocytophilum* HZ (APH-HZ) MSP2 determined using the Predict Secondary Structure (PSIPRED v3.0) program** (http://bioinf.cs.ucl.ac.uk/psipred/).
(PDF)

Figure S5 **Putative secondary structure of *A. phagocytophilum* Webster (APH-Webster) MSP2 determined using the Predict Secondary Structure (PSIPRED v3.0) program** (http://bioinf.cs.ucl.ac.uk/psipred/).
(PDF)
