# Supplementary material for: Molecular Characterization of msp2/p44 of Anaplasma phagocytophilum Isolated from Infected Patients and Haemaphysalis longicornis in Laizhou Bay, Shandong Province, China
Source: PLoS One. 2013 Oct 22;8(10):e78189. doi: 10.1371/journal.pone.0078189 (PMC3805589; doi:10.1371/journal.pone.0078189)
Supplement: Figure S1 — msp2 linear alignment of LZ-HGA-Agent, A. phagocytophilum HZ and A. phagocytophilum Webster at the nucleotide level. The alignment report was performed using the MegAlign program of the DNASTAR package. The nucleotide sequence names are indicated to the left, and the nucleotide numbers are shown to the right. The solid, deep red letters differ from the consensus, whereas all others match the consensus. APH-Webster: A. phagocytophilum Webster strain, APH-HZ: A. phagocytophilum HZ strain. (PDF) [file pone.0078189.s001.pdf]

Alignment Report of Untitled ClustalW (Slow/Accurate, IUB)

Sunday, November 18, 2012 05:57 PM

Majority	ATGAGAAAAGGAAAGATAATCTTAGGAAGCGTAATGATGTCGATGGCTATAGTCATGGCTGGGAATGATGTCAGGGCTCA	
	10 20 30 40 50 60 70 80	
LZ-HGA-Agent_msp2	ATGAGAAAAGGAAAGATAATCTTAGGAAGCGTAATGATGTCGATGGCTATAGTCATGGCTGGGAATGATGTCAGGGCTCA	80
APH-Webster_msp2	ATGAGAAAAGGAAAGATAATCTTAGGAAGCGTAATGATGTCGATGGCTATAGTCATGGCTGGGAATGATGTCAGGGCTCA	80
APH-HZ_msp2	--	0
Majority	TGATGACGTTAGCGCTTTGGAXACTGGTGGXGCGGGATATTTCTATGTTGGXTTGGATTACAGTCCAGCGTTTAGCAAGA	
	90 100 110 120 130 140 150 160	
LZ-HGA-Agent_msp2	TGATGACGTTAGCGCTTTGGATACTGGTGGAGCGGGATATTTCTATGTTGGCTTGGATTACAGTCCAGCGTTTAGCAAGA	160
APH-Webster_msp2	TGATGACGTTAGCGCTTTGGAGACTGGTGGTGCGGGATATTTCTATGTTGGTTTGGATTACAGTCCAGCGTTTAGCAAGA	160
APH-HZ_msp2	--	0
Majority	TAAGAGATTTTAGTATAAGGGAGAGTAACGGAGAGACXAAGGCAGTATATCCATACTTAAAGGATGGAAAGAGTGTAAAG	
	170 180 190 200 210 220 230 240	
LZ-HGA-Agent_msp2	TAAGAGATTTTAGTATAAGGGAGAGTAACGGAGAGACGAAGGCAGTATATCCATACTTAAAGGATGGAAAGAGTGTAAAG	240
APH-Webster_msp2	TAAGAGATTTTAGTATAAGGGAGAGTAACGGAGAGACTAAGGCAGTATATCCATACTTAAAGGATGGAAAGAGTGTAAAG	240
APH-HZ_msp2	-----------ATGATTAGGCCTTTGGGCATGGGAAT--AGTTGTTATATTTGTACT-----ACTTGGTACAGCGGTAAC	62
Majority	CTXGAGTCAXACAAGTTTGACTGGAACACACCTGATCCTCGGATTGGGTTTAAGGACAACATGCTTGTAGCTATGGAAGG	
	250 260 270 280 290 300 310 320	
LZ-HGA-Agent_msp2	CTTGAGTCACACAAGTTTGACTGGAACACACCTGATCCTCGGATTGGGTTTAAGGACAACATGCTTGTAGCTATGGAAGG	320
APH-Webster_msp2	CTAGAGTCTAACAAGTTTGACTGGAACACACCTGATCCTCGGATTGGGTTTAAGGACAACATGCTTGTAGCTATGGAAGG	320
APH-HZ_msp2	ATCGGCACATGCAGATAATGACAAGTCAGGCTTTTTGTGGGCTTGGGAT-ATGGAC--TATCCGTG-AGCCAGGTACA-	137
Majority	CAGTGTTGGTTATGGTATTGGTGGTGCCAGGGTTGAGCTTGAGATTGGTTACGAGCGCTTCAAGACCAAGGGTATTAGAG	
	330 340 350 360 370 380 390 400	
LZ-HGA-Agent_msp2	CAGTGTTGGTTATGGTATTGGTGGTGCCAGGGTTGAGCTTGAGATTGGTTACGAGCGCTTCAAGACCAAGGGTATTAGAG	400
APH-Webster_msp2	CAGTGTTGGTTATGGTATTGGTGGTGCCAGGGTTGAGCTTGAGATTGGTTACGAGCGCTTCAAGACCAAGGGTATTAGAG	400
APH-HZ_msp2	------TAATTTTAAAATCGATGATGGCGGAGAGACGC----G-TTGGCTGTTTCCCTTTCATGAGCGAGTGCACAGAGA	206
Majority	ATAGTGGTAGTAAGGAAGATGXAGCTGATACAGTATATCTACTAGCTAAGGAGTTAGCTTATGATGTTGTTACTGGACAG	
	410 420 430 440 450 460 470 480	
LZ-HGA-Agent_msp2	ATAGTGGTAGTAAGGAAGATGGAGCTGATACAGTATATCTACTAGCTAAGGAGTTAGCTTATGATGTTGTTACTGGACAG	480
APH-Webster_msp2	ATAGTGGTAGTAAGGAAGATGAAGCTGATACAGTATATCTACTAGCTAAGGAGTTAGCTTATGATGTTGTTACTGGACAG	480
APH-HZ_msp2	GGAATTGCACTCTGCAAATTT---CTATTGGGGTCCAGAGGTTGCTTCGGAGATAAGATTTCAGAGAGGTAACACTACTT	283
Majority	ACTGATAAXCTTXCXGCTGCTCTTGCTAAGACCTCXGGTAAGGAXTTTGTCCAGTTTGCTAAGGCGGTTGGGGTTTCXCA	
	490 500 510 520 530 540 550 560	
LZ-HGA-Agent_msp2	ACTGATAACCTTGCCGCTGCTCTTGCTAAAACCTCGGGGAAGGACTTTGTCCAGTTTGCTAAGGCGGTTGGGGTTTCCCA	560
APH-Webster_msp2	ACTGATAAGCTTACTGCTGCTCTTGCCAAGACCTCTGGTAAAGATATTGTTCAGTTTGCTAAGGCGGTTGGGGTTTCTCA	560
APH-HZ_msp2	TCGGGGGAGTGCTGGATATTTATTCTCTGCTGTACGGTTAGAAATTGATCTTACACACGAAAG-ATCCGAAATTCTAAA	362
Majority	XCCTGGXATTGATXXGAAGGTTTGTAAXGGGGGTXATGXACXXXGTAXAXXAA-XGAAGATAAXXGCCXACGG---GXXX	
	570 580 590 600 610 620 630 640	
LZ-HGA-Agent_msp2	CCCTACCATTGATGGGAAGGTTTGTAGGACGAAGAATGGACATAGTACACCAA-CGACGTTAACTGCCTACGGTAAGTAC	639
APH-Webster_msp2	TCCCGGTATTGATAAGAAGGTTTGTGATGGGGGTCATGCACGGGGAAAAAAGAGTGGAGATAATGGCTCACTG---GCCG	637
APH-HZ_msp2	ATCTGGGTTACAT---AAGGGCCGCAAAGGTGGTGGTATGCCCTTTGTATTAG--GAAAACACAAAGCGATGG-------	430
Majority	XCTGTA----CXGATGTAXAGACGGGAXAXAAXAATAATGXTGCACTXTGXAGTGGTXCGGGXXXAACXGXTAXAGCTGG	
	650 660 670 680 690 700 710 720	
LZ-HGA-Agent_msp2	GCTGTAGAGTCAGACGTAAAGACAGGAAACAACAATAATGTTGCACTATGCGGTGGTGCGGGGTCAACGGATGGAACTGG	719
APH-Webster_msp2	ACTATA----CGGATGGTGGCGCGTCACAGACGAATAAGACGGCTCAGTGTAGTGGTATGGG---AACCGGCAAAGCCGG	710
APH-HZ_msp2	--TGTC-------TT-TACAGAGGGGCTATGATCGTATTGATTTAATCGGAAGTCTTTCGCGTGAAAATGTTATCGCTAT	500
Majority	XAAXAGATXAXTGXXGGCTTXACX-XGAXTTXATTAACXXAACAAAGTTTGGAGAXXGAAGTAAGAAXTGGCCAACGGXG	
	730 740 750 760 770 780 790 800	
LZ-HGA-Agent_msp2	GAGTAGCTCACCGCAGGTTTTACG-CGACTTCATTAATGCCACAATGTTGGGAGATGGAAGTAAAAACTGGCCTACGTCG	798
APH-Webster_msp2	CAAGAGAGGATTG--GGCTTGAC--TGAGTTTGTTAACAAAACAAAGGTTGGAGA---AGGTAAGAATTGGCCAACGGGG	783
APH-HZ_msp2	AGAAAAATACATGGTGGCAGAACTAGGATATGATCAACTGAGAAGACTTTCGGTAATGCAGGAAGAAGAACTCAAGAGAG	580
Majority	-ACXTTAATGAXGGXXAXAACGTTAATGGXCX--CGCCTXTACX-AAXGATAACGCCGAAGCCGTAGCTAAAGACCTAGT	
	810 820 830 840 850 860 870 880	
LZ-HGA-Agent_msp2	-ACCTTAAAGGCGG--GTGGCTCAAATGGACCAACGCCTGTACATAACGACAACGCCAAAGCCGTAGCCAAAGACCTAGT	875
APH-Webster_msp2	TACGTTAATGATGGCGACAACGTTAATGTGCT--CGGCGATACG-AATGGTAACGCCGAAGCCGTAGCTAAAGACCTAGT	860
APH-HZ_msp2	----TTAATAAAACTAAGAAAGTTGTTGG--G---GCTTTTCCC-AGAAATAGGAGCGATAATTTTTTTAAT--T----T	644
Majority	ACAGGAGCTXACCCCTGAAGAAAAAACCATAGTAGCAGGGTTACTAGCTAAGACTATTGAAGGGGGTGAAGTTGTTGAGA	
	890 900 910 920 930 940 950 960	
LZ-HGA-Agent_msp2	ACAGGAGCTCACCCCCGAAGAAAAAACCATAGTAGCAGGGTTACTAGCCAAAACTATTGAAGGGGGTGAGGTTGTTGAAA	955
APH-Webster_msp2	ACAGGAGCTAACCCCTGAAGAAAAAACCATAGTAGCAGGGTTACTAGCTAAGACTATTGAAGGGGGTGAAGTTGTTGAGA	940
APH-HZ_msp2	ACTGGATCTGATGATTGTGCAAAGCGTTCTTTTCACAAAAGCTCTTGCTTTGACGGTTGAGGGTGCTGAAGTGATAGAGA	724
Majority	TXAGGGCGGTTTCTTCTACTTCTGTXATGGTXAATGCTTGTTATGATCTTCTTAGTGAAGGGTTAGGTG---------TT	
	970 980 990 1000 1010 1020 1030 1040	
LZ-HGA-Agent_msp2	TAAGGGCGGTTTCTTCTACTTCTGTGATGGTTAATGCTTGTTATGATCTTCTTAGTGAAGGGTTAGGTG---------TT	1026
APH-Webster_msp2	TCAGGGCGGTTTCTTCTACTTCCGTAATGGTCAATGCTTGTTATGATCTTCTTAGTGAAGGTTTAGGTG---------TT	1011
APH-HZ_msp2	TTATGGCCATAAGGAATACCACTGCTACGTTGAATTTGTGTTATGACTTTCCTAGCATGGAGTTGGTTAAACTCAACATT	804
Majority	GTTCCTTATGCTTGTGTTGGTCTTGGXGGTAACTTCGTGGGCGTTGTTGATGGGCACATCACTCCTAAGCTTGCTTATAG	
	1050 1060 1070 1080 1090 1100 1110 1120	
LZ-HGA-Agent_msp2	GTTCCTTATGCTTGTGTTGGTCTTGGGGGTAACTTCGTGGGCGTTGTTGATGGGCACATCACTCCTAAGCTTGCTTATAG	1106
APH-Webster_msp2	GTTCCTTATGCTTGTGTTGGTCTTGGCGGTAACTTCGTGGGCGTGGTTGATGGCCATATCACTCCTAAGCTTGCTTATAG	1091
APH-HZ_msp2	TCACCTTACACATGCGCCGGAATAGGTGGAAGCGTTATAGGTATTACAAAAGGGCACGCCAATTTACAACTTTCATACAA	884
Majority	ATTAAAGGCTGGXTTGAGTTATCAGCTTTCTCCTGAAATCTCTG-CTTTTGCGGGTGGATTCTACCATCGCGTTGTGGGA	
	1130 1140 1150 1160 1170 1180 1190 1200	
LZ-HGA-Agent_msp2	ATTAAAGGCTGGCTTGAGTTATCAGCTTTCTCCTGAAATCTCTG-CTTTTGCGGGTGGATTCTACCATCGCGTTGTGGGA	1185
APH-Webster_msp2	ATTAAAGGCTGGGTTGAGTTATCAGCTCTCTCCTGAAATCTCCG-CTTTTGCGGGTGGATTCTACCATCGCGTTGTGGGA	1170
APH-HZ_msp2	GCTCAAACTTGGTTTAAATTACC-GTTTCCTCTCAAATGCTGTGGCATATATAGGTACTTCATATCAGAAAGTTTTAGG-	962
Majority	GATGGCGTTTATGATGATCTGCCGGCTCAACGTCTTGTAGATGATACTAGTCCGGCGGGTCGTACTAAGGAXACTGCTAT	
	1210 1220 1230 1240 1250 1260 1270 1280	
LZ-HGA-Agent_msp2	GATGGCGTTTATGATGATCTGCCGGCTCAACGTCTTGTAGATGATACTAGTCCGGCGGGTCGTACTAAGGACACTGCTAT	1265
APH-Webster_msp2	GATGGCGTTTATGATGATCTGCCGGCTCAACGTCTTGTAGATGATACTAGTCCGGCGGGTCGTACTAAGGATACTGCTAT	1250
APH-HZ_msp2	GTCGGAGT--ACTATAATGTGCCACTAAAGCGGTTGGTTGACGATATCAGCCCAACCAACAGCGTTAGAGAGAAAACGAG	1040
Majority	TGCTAACTTCTCCATGGCTTATGTCGGTGGGGAATTTGGTGTTAGGTTCGCTTTTTAA	
	1290 1300 1310 1320 1330	
LZ-HGA-Agent_msp2	TGCTAACTTCTCCATGGCTTATGTCGGTGGGGAATTTGGTGTTAGGTTCGCTTTTTAA	1323
APH-Webster_msp2	TGCTAACTTCTCCATGGCTTATGTCGGTGGGGAATTTGGTGTTAGGTTCGCTTTTTAA	1308
APH-HZ_msp2	TGTGGGGTTTGGATTACAGTACGTTGGGTTAGAATTGGGCGCACGTGTATCTTTCTGA	1098

Decoration 'Decoration #1': Shade (with solid deep red) residues that differ from the Consensus.
