# Supplementary material for: Molecular Characterization of msp2/p44 of Anaplasma phagocytophilum Isolated from Infected Patients and Haemaphysalis longicornis in Laizhou Bay, Shandong Province, China
Source: PLoS One. 2013 Oct 22;8(10):e78189. doi: 10.1371/journal.pone.0078189 (PMC3805589; doi:10.1371/journal.pone.0078189)
Supplement: Figure S2 — MSP2 linear alignment of LZ-HGA-Agent, A. phagocytophilum HZ and A. phagocytophilum Webster at the amino acid level. The alignment report was performed using the MegAlign program of the DNASTAR package. Amino acid sequence names are indicated to the left, and the amino acid numbers are shown to the right. The amino acid residues colored a solid, deep red differ from the consensus sequence, and all others match the consensus. AA: amino acid residue. APH-Webster: A. phagocytophilum Webster strain, APH-HZ: A. phagocytophilum HZ strain. (PDF) [file pone.0078189.s002.pdf]

```
Majority              MRKGKIILGSVMMSMAIVMAGNDVRAHDDVSALDTGGAGYFYVGLDYSPAFSKIRDFSIRESNGETKAVYPYLKDGKSVK
                               10        20        30        40        50        60        70        80
LZ-HGA-Agent_MSP2_AA  MRKGKIILGSVMMSMAIVMAGNDVRAHDDVSALDTGGAGYFYVGLDYSPAFSKIRDFSIRESNGETKAVYPYLKDGKSVK  80
APH-Webster_MSP2_AA   MRKGKIILGSVMMSMAIVMAGNDVRAHDDVSALETGGAGYFYVGLDYSPAFSKIRDFSIRESNGETKAVYPYLKDGKSVK  80
APH-HZ_MSP2_AA        ------MIRPLGMGIVVIFVLLGTAVTSAHADNDKSG---FFVGLGYGLSVSQVHNFKIDDG-GETRWLFPFHERVHREE  70

Majority              LESXKFDWN-TPDPRIGFKDNMLVAMEGSVGYGIGGARVELEIGYERFKTKGIRDSGSKEDXADTVYLLAKELAYDVVTG
                               90       100       110       120       130       140       150       160
LZ-HGA-Agent_MSP2_AA  LESHKFDWN-TPDPRIGFKDNMLVAMEGSVGYGIGGARVELEIGYERFKTKGIRDSGSKEDGADTVYLLAKELAYDVVTG  159
APH-Webster_MSP2_AA   LESNKFDWN-TPDPRIGFKDNMLVAMEGSVGYGIGGARVELEIGYERFKTKGIRDSGSKEDEADTVYLLAKELAYDVVTG  159
APH-HZ_MSP2_AA        LHSANFYWGPEVASEIRFQR-GNTTFGGSAGYLFSAVRLEIDLTHERSE---ILKSGLHKGRKGGMPFVLGKHKAMVSL  146

Majority              QTDXLXAALAKTSGKDXVQFAKAVGVSHPGIDXKVC--XXGHXXXXXXXXXGXLAVXXDXXXXXXNKTAXCXGAGX-XGX
                              170       180       190       200       210       220       230       240
LZ-HGA-Agent_MSP2_AA  QTDNLAAALAKTSGKDFVQFAKAVGVSHPTIDGKVCRTKNGHSTPTTLTAYGKYAVESDVKTGNNNNVALCGGAGSTDGT  239
APH-Webster_MSP2_AA   QTDKLTAALAKTSGKDIVQFAKAVGVSHPGIDKKVC--DGGHARGKKSGDNGSLADYTDGGASQTNKTAQCSGMG--TGK  235
APH-HZ_MSP2_AA        QRGYDRIDLIGSLSRENVIAIEKYMVAELGYDQLRR-----------------LSVMQEEELKRVNKTKKVVGAFP----  205

Majority              XXXXXXXLXXFXNXTXXGXG-KNWPTXXXNXGXXXXXXXXXNXNAXAVAKDLVQELTPEEKTIVAGLLAKTIEGGEVVEI
                              250       260       270       280       290       300       310       320
LZ-HGA-Agent_MSP2_AA  GSSSPQVLRDFINATMLGDGSKNWPTSTLKAGGSNGPTPVHNDNAKAVAKDLVQELTPEEKTIVAGLLAKTIEGGEVVEI  319
APH-Webster_MSP2_AA   AGKRGLGLTEFVNKTKVGEG-KNWPTGYVNDGDNVNVLGDTNGNAEAVAKDLVQELTPEEKTIVAGLLAKTIEGGEVVEI  314
APH-HZ_MSP2_AA        ---------------------RNRSDNFFN---------------------LL-DLMIVQSVLFTKALALTVEGAEVIEI  242

Majority              RAVSSTSVMVNACYDLLSEG---LGVVPYACVGLGGNFVGVVDGHITPKLAYRLKAGLSYQLSPEISAFAGGFYHRVVGD
                              330       340       350       360       370       380       390       400
LZ-HGA-Agent_MSP2_AA  RAVSSTSVMVNACYDLLSEG---LGVVPYACVGLGGNFVGVVDGHITPKLAYRLKAGLSYQLSPEISAFAGGFYHRVVGD  396
APH-Webster_MSP2_AA   RAVSSTSVMVNACYDLLSEG---LGVVPYACVGLGGNFVGVVDGHITPKLAYRLKAGLSYQLSPEISAFAGGFYHRVVGD  391
APH-HZ_MSP2_AA        MAIRNTTATLNLCYDFPSMELVKLNISPYTCAGIGGSVIGITKGHANLQLSYKLKLGLNYRFLSNAVAYIGTSYQKVLGS  322

Majority              GVYDDLPAQRLVDDTSPAGRTKDTAIANFSMAYVGGEFGVRFAF-
                              410       420       430       440
LZ-HGA-Agent_MSP2_AA  GVYDDLPAQRLVDDTSPAGRTKDTAIANFSMAYVGGEFGVRFAF.                                    441
APH-Webster_MSP2_AA   GVYDDLPAQRLVDDTSPAGRTKDTAIANFSMAYVGGEFGVRFAF.                                    436
APH-HZ_MSP2_AA        -EYYNVPLKRLVDDISPTNSVREKTSVGFGLQYVGLELGARVSF.                                    366
```

Decoration 'Decoration #1': Shade (with solid deep red) residues that differ from the Consensus.
