# Supplementary material for: Molecular Characterization of msp2/p44 of Anaplasma phagocytophilum Isolated from Infected Patients and Haemaphysalis longicornis in Laizhou Bay, Shandong Province, China
Source: PLoS One. 2013 Oct 22;8(10):e78189. doi: 10.1371/journal.pone.0078189 (PMC3805589; doi:10.1371/journal.pone.0078189)
Supplement: Figure S3 — Putative secondary structure of LZ-HGA-Agent MSP2 determined using the Predict Secondary Structure (PSIPRED v3.0) program (http://bioinf.cs.ucl.ac.uk/psipred/). (PDF) [file pone.0078189.s003.pdf]

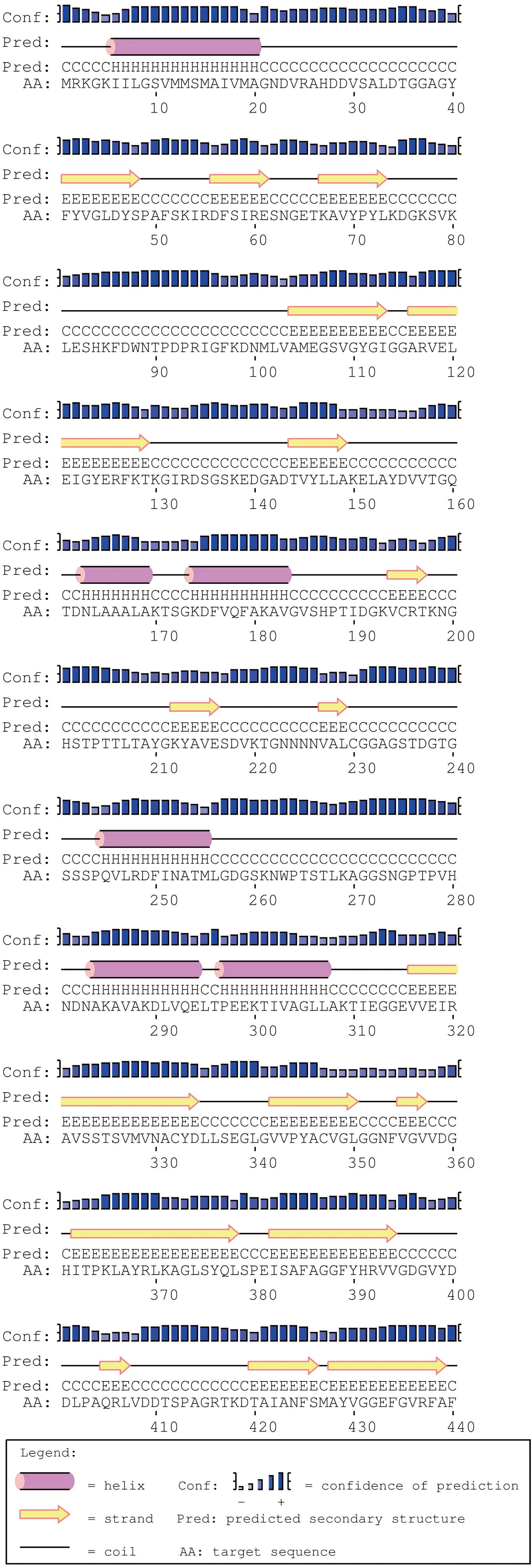
Conf:
Pred:
Pred: CCCCCHHHHHHHHHHHHHHHCCCCCCCCCCCCCCCCCCCC
AA: MRKGKIILGSVMMSMAIVMAGNDVRAHDDVSALDTGGAGY
10 20 30 40
Conf:
Pred:
Pred: EEEEEEEECCCCCCCEEEEEECCCCCEEEEEEEECCCCCCC
AA: FYVGLDYSPAFSKIRDFSIRESNGETKAVYPYLKDGKSVK
50 60 70 80
Conf:
Pred:
Pred: CCCCCCCCCCCCCCCCCCCCCCCCEEEEEEEEEEECCEEEEE
AA: LESHKFDWNTPDPRIGFKDNMLVAMEGSVGYGIGGARVEL
90 100 110 120
Conf:
Pred:
Pred: EEEEEEEEECCCCCCCCCCCCCCEEEEEECCCCCCCCCCC
AA: EIGYERFKTKGIRDSGSKEDGADTVYLLAKELAYDVVTGQ
130 140 150 160
Conf:
Pred:
Pred: CCHHHHHHHCCCCHHHHHHHHHHCCCCCCCCCCEEEEECCC
AA: TDNLAAALAKTSGKDFVQFAKAVGVSHPTIDGKVCRTKNG
170 180 190 200
Conf:
Pred:
Pred: CCCCCCCCCCCEEEEECCCCCCCCCCEEECCCCCCCCCCC
AA: HSTPTTLTAYGKYAVESDVKTGNNNNVALCGGAGSTDGTG
210 220 230 240
Conf:
Pred:
Pred: CCCCHHHHHHHHHHHCCCCCCCCCCCCCCCCCCCCCCCCC
AA: SSSPQVLRDFINATMLGDGSKNWPTSTLKAGGSNGPTPVH
250 260 270 280
Conf:
Pred:
Pred: CCCHHHHHHHHHHHCCHHHHHHHHHHHCCCCCCCCEEEEE
AA: NDNAKAVAKDLVQELTPEEKTIVAGLLAKTIEGGEVVEIR
290 300 310 320
Conf:
Pred:
Pred: EEEEEEEEEEEEEECCCCCCCEEEEEEEEEECCCCEEECCC
AA: AVSSTSVMVNACYDLLSEGLGVVPYACVGLGGNFVGVVDG
330 340 350 360
Conf:
Pred:
Pred: CEEEEEEEEEEEEEEEEECCCEEEEEEEEEEEEEECCCCCC
AA: HITPKLAYRLKAGLSYQLSPEISAFAGGFYHRVVGDGVYD
370 380 390 400
Conf:
Pred:
Pred: CCCCEEECCCCCCCCCCCCEEEEEEEECEEEEEEEEEEEEC
AA: DLPAQRLVDDTSPAGRTKDTAIANFSMAYVGGEFGVRFAF
410 420 430 440
Legend:
= helix
Conf: = confidence of prediction
- +
= strand
Pred: predicted secondary structure
= coil
AA: target sequence
