# Supplementary material for: Molecular Characterization of msp2/p44 of Anaplasma phagocytophilum Isolated from Infected Patients and Haemaphysalis longicornis in Laizhou Bay, Shandong Province, China
Source: PLoS One. 2013 Oct 22;8(10):e78189. doi: 10.1371/journal.pone.0078189 (PMC3805589; doi:10.1371/journal.pone.0078189)
Supplement: Figure S4 — Putative secondary structure of A. phagocytophilum HZ (APH-HZ) MSP2 determined using the Predict Secondary Structure (PSIPRED v3.0) program (http://bioinf.cs.ucl.ac.uk/psipred/). (PDF) [file pone.0078189.s004.pdf]

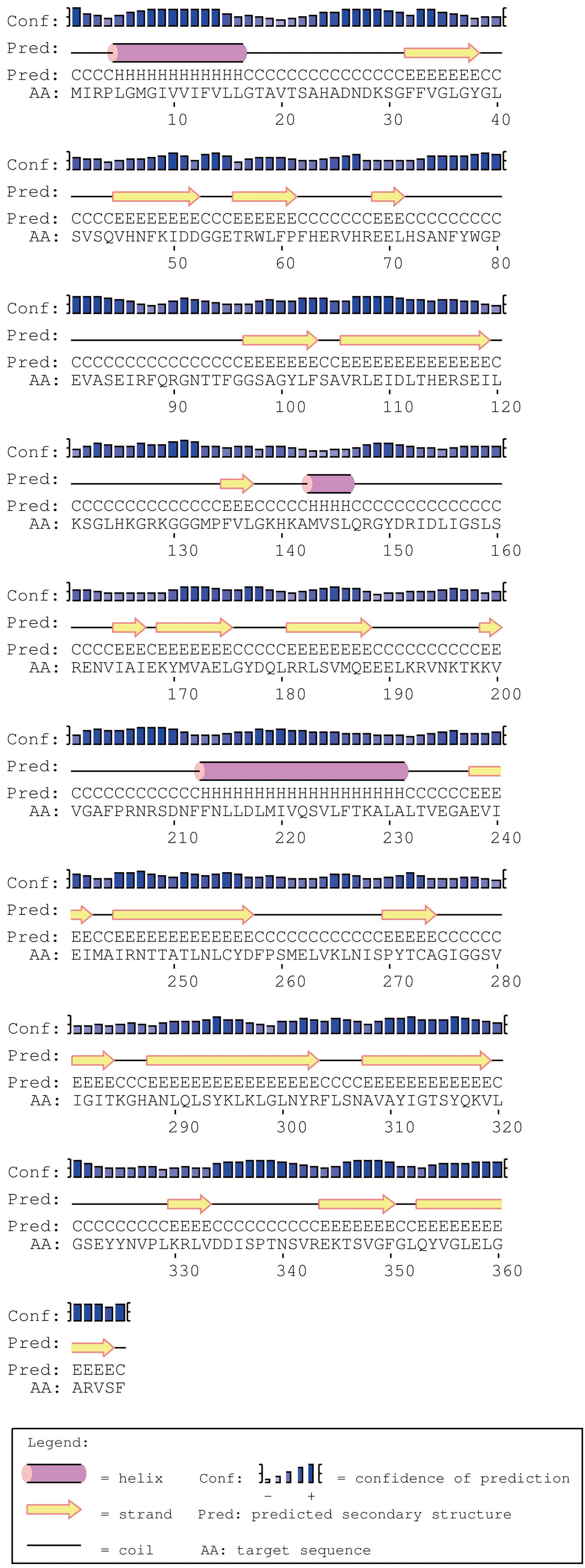
Conf:
Pred:
Pred: CCCCHHHHHHHHHHHHCCCCCCCCCCCCCCCEEEEEEECC
AA: MIRPLGMGIVVIFVLLGTAVTSAHADNDKSGFFVGLGYGL
10 20 30 40
Conf:
Pred:
Pred: CCCCEEEEEEEEECCCEEEEEEECCCCCCCEEECCCCCCCCC
AA: SVSQVHNFKIDDGGETRWLFPFHERVHREELHSANFYWGP
50 60 70 80
Conf:
Pred:
Pred: CCCCCCCCCCCCCCCCEEEEEEECCEEEEEEEEEEEEEEC
AA: EVASEIRFQRGNTTFGGSAGYLFSAVRLEIDLTHERSEIL
90 100 110 120
Conf:
Pred:
Pred: CCCCCCCCCCCCCCEEECCCCCHHHHCCCCCCCCCCCCCC
AA: KSGLHKGRKGGGMPFVLGKHKAMVSLQRGYDRIDLIGSLS
130 140 150 160
Conf:
Pred:
Pred: CCCCEEECEEEEEEECCCCCEEEEEEEECCCCCCCCCCEE
AA: RENVIAIEKYMVAELGYDQLRRLSVMQEEELKRVNKTKKV
170 180 190 200
Conf:
Pred:
Pred: CCCCCCCCCCCCHHHHHHHHHHHHHHHHHHHCCCCCCEEE
AA: VGAFPRNRSDNFFNLLDLMIVQSVLFTKALALTVEGAEVI
210 220 230 240
Conf:
Pred:
Pred: EECCEEEEEEEEEEEEECCCCCCCCCCCCEEEEEECCCCC
AA: EIMAIRNTTATLNLCYDFPSMELVKLNISPYTCAGIGGSV
250 260 270 280
Conf:
Pred:
Pred: EEEECCCEEEEEEEEEEEEEEEEECCCCEEEEEEEEEEEEEC
AA: IGITKGHANLQLSYKLKLGLNYRFLSNAVAYIGTSYQKVL
290 300 310 320
Conf:
Pred:
Pred: CCCCCCCCCEEEECCCCCCCCCCCEEEEEEEECCEEEEEEEE
AA: GSEYYNVPLKRLVDDISPTNSVREKTSVGFGLQYVGLELG
330 340 350 360
Conf:
Pred:
Pred: EEEEC
AA: ARVSF
Legend:
= helix
Conf:
= confidence of prediction
–
+
= strand
Pred: predicted secondary structure
= coil
AA: target sequence
