# Supplementary material for: Molecular Characterization of msp2/p44 of Anaplasma phagocytophilum Isolated from Infected Patients and Haemaphysalis longicornis in Laizhou Bay, Shandong Province, China
Source: PLoS One. 2013 Oct 22;8(10):e78189. doi: 10.1371/journal.pone.0078189 (PMC3805589; doi:10.1371/journal.pone.0078189)
Supplement: Figure S5 — Putative secondary structure of A. phagocytophilum Webster (APH-Webster) MSP2 determined using the Predict Secondary Structure (PSIPRED v3.0) program (http://bioinf.cs.ucl.ac.uk/psipred/). (PDF) [file pone.0078189.s005.pdf]

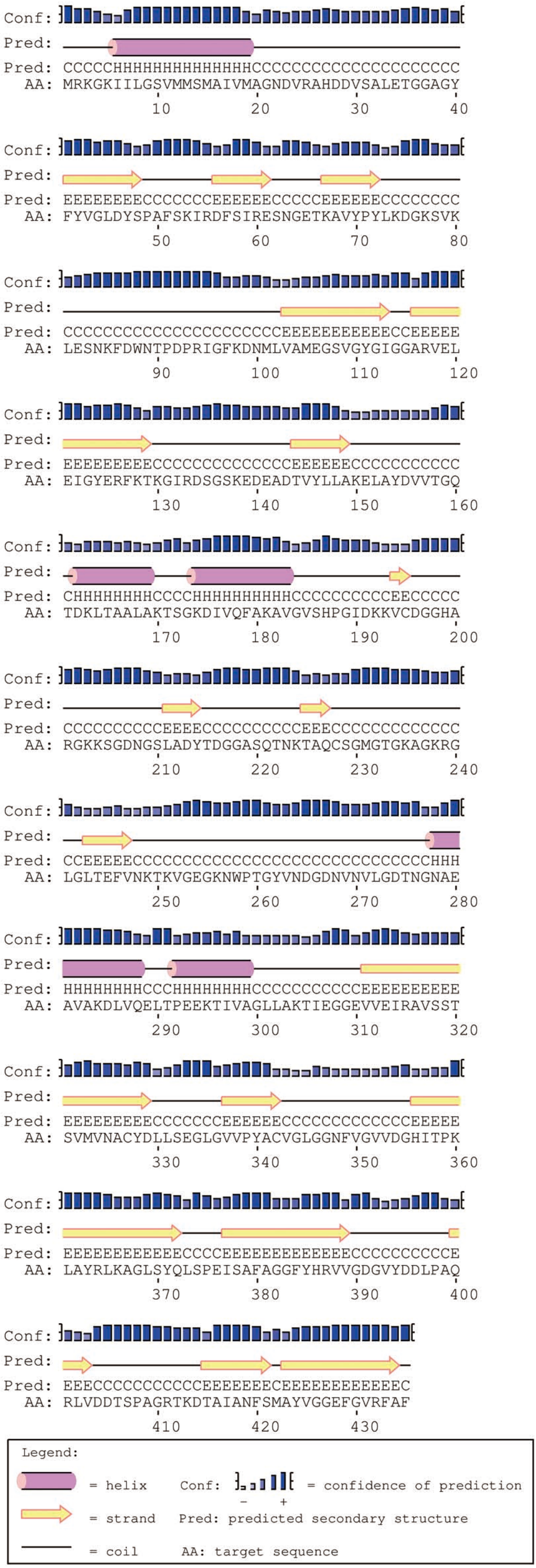

Conf:
Pred:
Pred: CCCCCHHHHHHHHHHHHHHCCCCCCCCCCCCCCCCCCCCC
AA: MRKGKIILGSVMMSMAIVMAGNDVRAHDDVSALETGGAGY
10 20 30 40
Conf:
Pred:
Pred: EEEEEEEECCCCCCCEEEEEEECCCCCEEEEEEECCCCCCCC
AA: FYVGLDYSPAFSKIRDFSIRESNGETKAVYPYLKDGKSVK
50 60 70 80
Conf:
Pred:
Pred: CCCCCCCCCCCCCCCCCCCCCCCEEEEEEEEEEEECCEEEEE
AA: LESNKFDWNTPDPRIGFKDNMLVAMEGSVGYGIGGARVEL
90 100 110 120
Conf:
Pred:
Pred: EEEEEEEEECCCCCCCCCCCCCCEEEEEEECCCCCCCCCC
AA: EIGYERFKTKGIRDSGSKEDEADTVYLLAKELAYDVVTGQ
130 140 150 160
Conf:
Pred:
Pred: CHHHHHHHHCCCCHHHHHHHHHHCCCCCCCCCCEECCCCC
AA: TDKLTAALAKTSGKDIVQFAKAVGVSHPGIDKKVCDGGHA
170 180 190 200
Conf:
Pred:
Pred: CCCCCCCCCCEEEECCCCCCCCCCEEECCCCCCCCCCCCC
AA: RGKKSGDNGSLADYTDGGASQTNKTAQCSGMGTGKAGKRG
210 220 230 240
Conf:
Pred:
Pred: CCEEEEEECCCCCCCCCCCCCCCCCCCCCCCCCCCCCHHH
AA: LGLTEFVNKTKVGEGKNWPTGYVNDGDNVNVLGDTNGNAE
250 260 270 280
Conf:
Pred:
Pred: HHHHHHHHCCCHHHHHHHHCCCCCCCCCCEEEEEEEEEE
AA: AVAKDLVQELTPEEKTIVAGLLAKTIEGGEVVEIRAVSST
290 300 310 320
Conf:
Pred:
Pred: EEEEEEEEECCCCCCCEEEEEECCCCCCCCCCCCCCEEEEE
AA: SVMVNACYDLLSEGLGVVPYACVGLGGNFVGVVDGHITPK
330 340 350 360
Conf:
Pred:
Pred: EEEEEEEEEEEECCCCEEEEEEEEEEEEEECCCCCCCCCCE
AA: LAYRLKAGLSYQLSPEISAFAGGFYHRVVGDGVYDDLPAQ
370 380 390 400
Conf:
Pred:
Pred: EEECCCCCCCCCCCCEEEEEEEECEEEEEEEEEEEEEC
AA: RLVDDTSPAGRTKDTAIANFSMAYVGGEFGVRFAF
410 420 430
Legend:
= helix
Conf: = confidence of prediction
- +
= strand
Pred: predicted secondary structure
= coil
AA: target sequence
